# Tuberculosis*Read before the American International Congress on Tuberculosis, New York, November 16, 1906.

**Published:** 1907-09

**Authors:** Moritz Benedikt

**Affiliations:** Vienna, Austria; Honorary Member of the Medico-Legal Society of New York


### Abstracts and Selections.

### Tuberculosis.*

BY PROFESSOR MORITZ BENEDIKT, M. D., VIENNA, AUSTRIA.
Honorary Member of the Medico-Legal Society of New York.

Every congress in medical science involves, as Virchow said, a new phase of error or errors. In the epoch in which the patho-

*Read before the American International Congress on Tuberculosis, New York, November 16, 1906.

logical anatomy was sovereign in medicine, one had forgotten the contagiousness of phthisis, though old Galen had taught it and knew well its conditions. When about twenty years ago Koch discovered the bacillus of tuberculosis, opinions changed rapidly and one supposed an exceeding degree of contagiousness, which became more fatal to the social position of the sick than to their health and to the health of persons surounding them. The demonstration of some such microbes in a sputum had the effect that the patient was avoided as one suffering from the plague.

It was forgotten at once the importance of predisposition and that millions and milliards of Koch's creatures can enter a body without doing any harm. The importance of heredity was entirely lost sight of, and one intended to cover all the countries of the earth with colonnades of spittoons, though we know now that the slightest and shortest sunshine and even diffused light destroy the microbes; and even fresh air during the night seems to have the same effect for a reason you will learn later.

It must be here asked if a sputum from which the bacillus Koch has been removed loses its toxic effect. In other words, we must ask if the sputum ceases to be a "virus" when free from the microbe. We have today no answer to this question, the less so as it is very probable that the sputum of the tuberculous is contagious only when they are already phthisic and probably by symbiosis with another microbe.

Hitherto, one has neglected the danger of infection arising from sweat, which is far greater than that of the sputum, as was already taught by Galen.

It is a general lack in pathology that we do not sufficiently study the part played by perspiration. This was done with exceeding zeal in former times.

It is probable that the old opinion that through perspiration a deal of *materia peccans* is evacuated from the body is correct. Also with the sweat of tuberculous is eliminated a good deal of special microbes, probably together with dangerous toxins.

As fever is the natural reaction against morbid irritations of the organs and an endeavor to save the infected, so sweat is a saving action against accumulation of noxious materia in the body. The feverish reaction may burn the body and exceeding sweat may melt the patient to total consumption.

We must regulate the one as well as the other process to prevent the saving action from killing.

This leads us to the question of hygeia of the tuberculous and phthisic individuals. The bathers all over the world may be divided into those who use or abuse hot and warm baths and those who use or abuse hydropathic proceedings. The hot and warm baths increase useful perspiration, but may eliminate the power of resistance against the evils of changing temperature and they may effeminate. We are sure today that refrigeration is far less dangerous for health than was believed in former time. The hydropathic proceeding strengthens the power of resistance against cold and increases the energy of the nervous system. An abuse of them may suppress the perspiration and certainly is not an indifferent factor for the nervousness of our epoch. The Nineteenth Century has offered us the benefit of hydrotherapy; the Twentieth must deliver us from the megalomania and from the pangragmasia of hydropaths.

The importance of applications of hot, tepid or cold for tuberculous patients is clear; the special indications the physician must find and adopt at every moment. Our care is today principally directed to tuberculosis of the lungs and of the respiratory tractus. The manner of treatment and of protection for tuberculosis of the glands, of the bones and of the peritoneum are very different from that of the respiratory organs. Also the tuberculous affections, the acute and the chronic of the brain demand other measures. We know that these cases have a great tendency to spontaneous sanation and their infectious power is dubious.

We will pass over all the forms except respiratory tuberculosis. Here we must principally from the point of view of protection and cure insist on the separation of tuberculosis from phthisis.

We will speak of tuberculosis of the lungs so long as we observe only apicitis and catarrhs and little disposition for hemoptysis. These cases may be designated as "slight" ones; we speak then of simple tuberculosis; when the sputum becomes purulent, when caverns appear and the danger of hemoptysis is great, when the patients become feverish and suffer from consummate sweat we may speak or "heavy" cases or of phthisis.

It is very probable that phthisis arises from smybiosis of the bacillus Koch with other microbes. I was the first, I believe, to insist on the dangerous symbiosis with the microbe of influenza. The purulent epoch of the illness depends on combination with a form of streptococcus or with a special microbe announced by Schroen.

For light cases we need no sanatoria. A well organized patronage can procure homes of recovery in places with suitable air and sunshine and protected from winds with the help of good food, naturally under medical surveyance of treatment, and of hygiene measures. Special care must be taken for the cleaning by hot air of the body linen and especially of the bed linen. In this way you have the possibility to avoid the crowding of patients and you can prevent a great increase of infection; f. e. by influenza. Such patients can be treated singly or in small numbers in family homes, f. e. in farmhouses. Bedrooms for every patient should be obligatory also in cases of simple tuberculosis. If you have made a mistake in the choice of places, you can correct it without great difficulty, and you can satisfy the need of change in the different seasons and at little expense you can protect a great number of those who require your protection. You must never forget that war against misery is the best means against the beginning and the development of tuberculosis, and of its progress to phthisis. The higher the standard of life of the poor, the better the sanitary statistics.

In quite a different manner must the phthisical be treated. A sanatorium for phthisical should be fitted up in more delicate manner than even a hospital for surgical patients. By antiseptic bandaging we can prevent contagion of the other patients. But we can not bandage the whole body and the lungs of the phthisical to prevent the infection by sweat or breath.

Therefore, the first condition for treating such patients is isolation, and not only through the night, but also through the day. It will not be advisable to crowd them in little pavilions.

It will be useful to construct these pavilions in such a way that sunshine can penetrate into both rooms, the bedroom and the room for sojourn through the day.

I take this opportunity to remark that we should not insist in all circumstances on open air treatment. In bad weather by means of French windows the apartment can not be exposed enough to open air, and it seems to be a perversity to apply the open air treatment absolutely and under all circumstances.

I now wish to call your attention to the so-called "model sanatoria." I know one situated in a beautiful valley amidst fine forests. If you saw this position on a fine spring or summer day you would be delighted with it. But during the winter and autumn months the country is enveloped in mist. At a distance of a few miles you find a site where there are few days in the year without sunshine in a higher mountain situation.

I have studied this spot from the topographical point of view in reference to tuberculosis. When already at a very short distance the fitness for the establishing of sanatoria is so different, you will conceive how easily mistakes arise. Such sanatoria founded by philanthropists are often obliged to falsify the statistics to maintain the favor of their patrons. They do so admitting principally light cases and dismissing the patients whose state had become worse and dangerous.

This leads us to the question of responsibility for prevention and cure of the scourge. The patronage of philanthropists can do much. But generally this aid is insufficient, and affords no guarantee that the aid will always be reliable, independent of the caprices and whims of philanthropists.

As a rule it must be recognized that the responsibility is shared by the whole community, i. e., by those representative authorities. These authorities may be different in different counties according to the political organization.

I come to the end of my statements. You know the different efforts made to create specific methods against tuberculosis. I believe that we have known the specific for more than two thousand years. It is the light of the sun penetrating fresh air. Since the time we left the theory of *emanati nofo* Newton and of the old philosophers, we have considered light as exclusively produced by the movement of ether and its effect on matter as dynamic or to use a neologism as catalytic.

This catalytic effect is either destructive decompositive in the way platina acts on certain chemical substances, or light works in an opposite manner synthetically, as we see f. e. in plants.

But today for us ether is no longer a product of our scientific imagination, but without doubt a matter of the nature of radium and its chemical cognates. We must suppose the light enters the different bodies and creatures introducing molecules of matter, which combine with the different matters. So in plants, f. e. arise colors, etc. We know as well, as I have already said, that air, penetrated by sunshine, is the specific against tuberculosis. The problem now is to separate the active parts of this air and give them the different forms, to make them active for the different internal.

I believe that this separation of the component part of the air will not be the only method to find the specifics. We shall probably find f. e. in plants such condition of the atmospheric specifics as will be directly fit to destroy the toxic matter in the different organs. It is possible that also the study and preparation of any serum will succeed. But I am of opinion that this way should not be adopted exclusively.

In conclusion allow me to draw a fancy picture. I know that in general men of science do not like fancies. But a prominent man like you knows very well the high importance of imagination for scientific creations, though Theseus in "Midsummer Nights Dream" forgot the part of imagination in science. But another authority as great as Shakespeare and a more competent one in scientific questions, Goethe, did not omit this important part and when Kant created a theory of descent and called it an "adventure of intellect" Goethe declared himself prepared for this "fancy" and this work of imagination became so serious for science.

Ever since we have known the part played by microbes in the etiology of tuberculosis, I have had the mental reservation that, perhaps, these microbes may be originally not a parasite, but a product of the illness, with the reserve, that these infectious creatures when they emigrate may become parasites in other individuals.

This idea occurred to me on consideration of the hereditary and almost sudden cases and of the presence of tuberculous microbes in lupus-knots. But I did not dare to give utterance to this fancy.

Today, however, we know through the researches of Traube, of Charles Vogt, Harting, von Schroen, Quincke, Leduc and the Mexican Herrera, that by the processes of precipitation and crystallization there arises organ and forms of cells without the intervention of any vital power. Today we can imagine that in the solution within the body there arise organic forms with vital qualities when vital matter and vital energies combine with the formative power of inorganic processes.—*Medico-Legal Journal,* June, 1907.

